# A Fright and Its Consequences

**Published:** 1888-11-24

**Authors:** 


### A FRIGHT AND ITS CONSEQUENCES.

A few weeks ago a girl of fourteen, of studious habits, was frightened by a man near Llandudno, and, in running away to escape from him, stumbled and fell. It is not stated that any evident injury was noticed at the time, but about a month later symptons of chorea, or St. Vitus's dance, began to show themselves. It was first noticed that she held her knife and fork awkwardly when eating. Then jerky movements, confined to one side, showed themselves; and finally the jerky movements became general. The convulsions in the course of seven or eight days grew exceedingly violent; all the limbs, as well as the head and trunk, were jerked about with such force that mechanical restraint became necessary to prevent the patient involuntarily throwing herself out of bed. By this time she had been removed to the infirmary at Liverpool. Remedies of the most powerful kind and in very large doses had to be given in order to produce cessation of the alarming symptoms and to procure sleep. Within another week a typhoid condition ensued. The patient became entirely unconscious, and passed all her motions in bed like a baby. The violent movements still continued when the mechanical restraint was intermitted, and the limbs presented numerous bruises and excoriations from the blows the child had given herself in her dreadful struggles. It is impossible to say certainly that the typhoid fever had its origin in the fright, though it may have been so. There need be no doubt, however, that the other symptoms were entirely due to the nervous shock brought on by fever. Women and girls are peculiarly sensitive to sudden alarms. The nervous system of the civilised female is a highly-delicate and sensitive organism. Teachers and nurses, as well as parents, should have a thoroughly intelligent knowledge of the nerves and the health, as well as of the mind and temper of their charges. It is a very rough-and-ready civilisation which takes no account of nerves and health in the training of the young, or in the general conduct of life. Instead of grudging the public a little physiological and medical knowledge, a humane science, such as medicine should pre-eminently be, ought to strive to make every human being thoroughly familiar with the general principles of physical and mental well-being. As civilisation advances the mind and the brain, with the whole nervous system, occupy a constantly-increasing space in the scheme of things. Medicine is bound, by the highest considerations of public and individual duty, to expand itself to meet the conditions of the times.

